# Non-linear association of triglyceride-glucose index with prevalence of prediabetes and diabetes: a cross-sectional study

**DOI:** 10.3389/fendo.2023.1295641

**Published:** 2023-12-13

**Authors:** Linhao Zhang, Ling Zeng

**Affiliations:** ^1^ Department of Critical Care Medicine, West China Hospital, Sichuan University, Chengdu, China; ^2^ West China School of Nursing, Sichuan University, Chengdu, China

**Keywords:** triglyceride-glucose index (TyG index), non-linear, prediabetes, diabetes, cross-sectional

## Abstract

**Background:**

The Triglyceride-glucose (TyG) index has been acknowledged as a convenient, cost-effective, and relatively simple marker for insulin resistance (IR). Meanwhile, prediabetes, described as an asymptomatic, moderately hyperglycemic state, tends to be more prevalent than diabetes. Thus, the objective of this study was to explore the relationship between the TyG index and the prevalence of both prediabetes and diabetes within the U.S. population.

**Methods:**

This study utilized a cross-sectional dataset derived from the National Health and Nutrition Survey (NHANES) spanning 1999 to 2018. The subjects were individuals aged 18 years and above, who had available fasting glucose and fasting triglyceride information, permitting a diagnosis of prediabetes or diabetes. The TyG index was computed using laboratory data, and participants were subsequently categorized into quartiles based on this information. The relationship between the TyG index and the prevalence of prediabetes and diabetes was investigated using logistic regression analysis.

**Results:**

Out of the 25,159 participants, 23.88% were found to have prediabetes, while 16.22% were diagnosed with diabetes. After adjusting for confounding factors, a linear increase in relative odds was observed in Q2 (OR: 1.69; 95% CI: 1.52, 1.89), Q3 (OR: 2.57; 95% CI: 2.30, 2.88), and Q4 (OR: 4.88; 95% CI: 4.33, 5.49) groups in comparison to the reference group, Q1. In addition, a non-linear relationship was observed between the TyG index and the prevalence of prediabetes and diabetes. Specifically, patients with a TyG index greater than 8.00 overall exhibited a significantly higher risk of prediabetes and diabetes, confirming that an increase in the TyG index is associated with a corresponding increase in risk. However, this shift showed gender-specific variations; the threshold was observed at 8.00 in males but shifted to 9.00 in females.

**Conclusion:**

The TyG index demonstrated a non-linear positive correlation with both prediabetes and diabetes. This suggests that maintaining the TyG index at a certain, reduced level could potentially aid in preventing the onset of prediabetes and diabetes.

## 1 Introduction

In 2025, diabetes is projected to have a global incidence of 26.6 million and a prevalence of 579.9 million (1). According to the World Health Organization (WHO), diabetes is projected to ascend to the seventh leading cause of death worldwide by 2030, a consequence of its escalating prevalence (2). Moreover, the population of individuals with prediabetes, a condition characterized by blood glucose levels that exceed the norm but fall below the diagnostic threshold for type 2 diabetes, is also anticipated to escalate (3). Projections estimate that by 2030, the number of individuals with prediabetes will surpass 470 million (4). Significantly, prediabetes should not be underestimated. Compared to individuals with normal glucose metabolism, those with prediabetes are linked with a heightened risk of cardiovascular disease and diabetic microangiopathy (5–7). Both diabetes and prediabetes are significantly influenced by insulin resistance (IR) and pancreatic beta cell dysfunction, elements that play paramount roles in their pathophysiology. The implementation of preventive strategies and effective treatment methods for diabetes and prediabetes is crucial, given the differing impacts of these factors on prediabetic subgroups across various races and ethnicities (8–11). As such, it is critical to implement preventive strategies and effective treatment methods for diabetes and prediabetes. This will help to effectively reduce the onset and progression of clinical complications and cardiovascular diseases (CVDs) associated with these conditions.

The triglyceride-glucose (TyG) index, a cost-effective and straightforward marker for insulin resistance (IR), is widely recognized as a convenient tool (12). It has been demonstrated to be associated with an elevated risk of CVD in the general population (13). Research conducted in Korea suggests that the TyG index could serve as a valuable predictor of CVD in individuals aged 40 and above, and also in younger adults aged 20-39 (14, 15). Previous research also suggests that the TyG index can forecast the risks of arteriosclerosis, coronary calcification, and diabetes (16–18). Interestingly, the number of individuals diagnosed with prediabetes significantly outnumbers those diagnosed with diabetes (19). Hence, it is vital to diagnose prediabetes and diabetes at an early stage for efficient management and prevention of disease progression (20, 21). While the association between the TyG index and prediabetes has been confirmed in Asian (22), adolescent (23), and elderly populations (24), its impact on prediabetes and diabetes in the general population remains underexplored. Specifically, there is a paucity of research probing more intricate aspects of this relationship, such as non-linear associations, as well as the identification of specific subgroups within the population that may be particularly susceptible. Moreover, a lack of large-scale studies impedes our understanding of the predictive value of the TyG index in relation to prediabetes and diabetes. In light of this, we conducted an extensive cross-sectional study to establish the association between the TyG index and prediabetes and diabetes in American adults. We sourced our information from the National Health and Nutrition Examination Survey (NHANES) for this study.

## 2 Methods

### 2.1 Study design and participants

The data employed in this study were retrieved from the NHANES database for the years 1999-2018. NHANES is an ongoing survey utilizing a complex, multi-step probability sampling method to select a representative sample of the United States population. The primary goal is to evaluate the health and dietary status of both adults and children living in the United States. The research protocol of NHANES has received approval from the Institutional Review Board of the National Center for Health Statistics (NCHS), and all study participants have given written informed consent. For more comprehensive information, please visit the website: www.cdc.gov/nchs/nhanes/irba98.htm.

**Figure 1:**
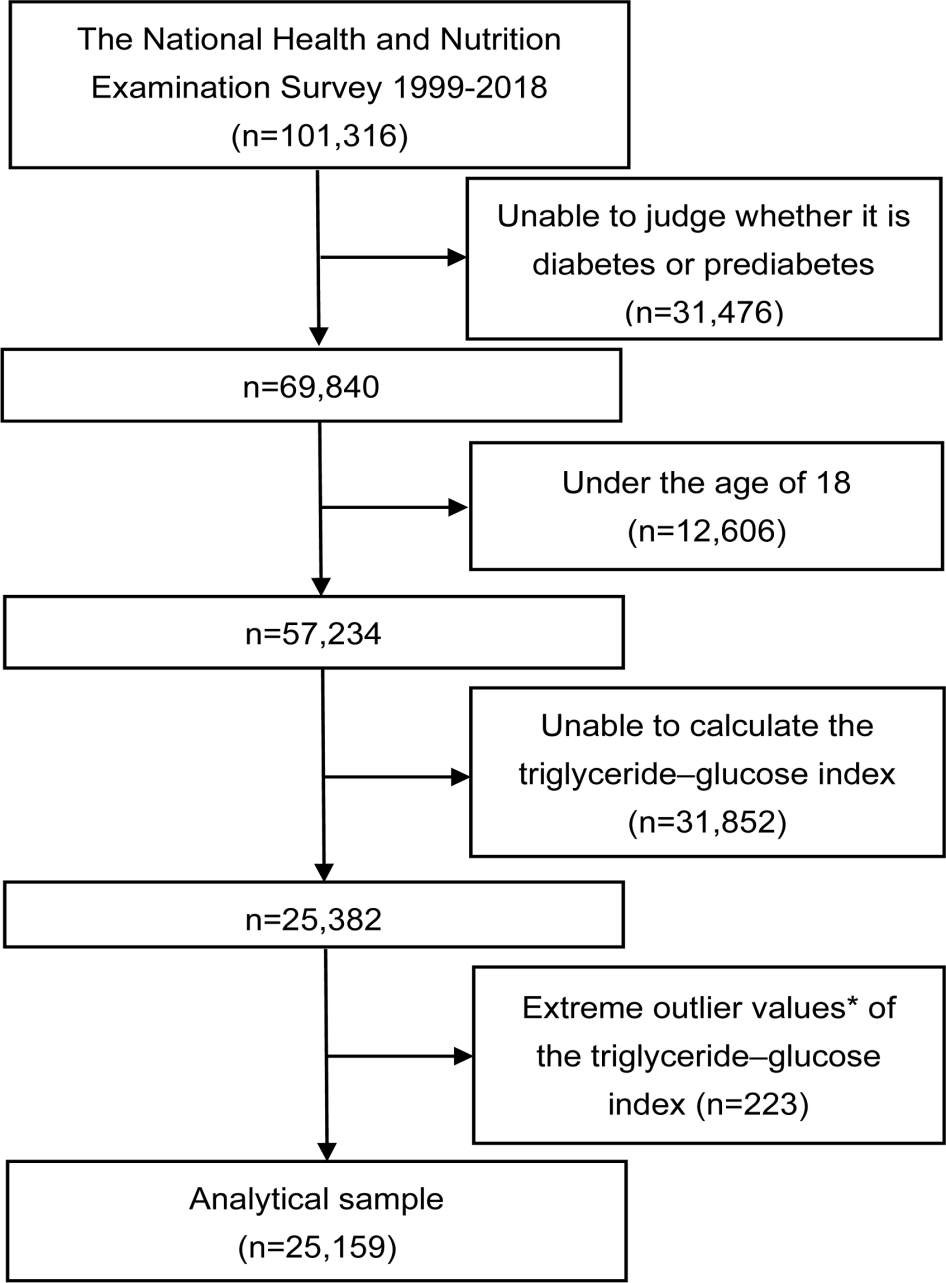
Flowchart of the study. *Extreme outlier values, defined as those over 3 standard deviations from the mean.

The data used in this cross-sectional study were collected from twelve consecutive cycles (1999–2018) of the NHANES database, with each cycle representing a two-year period. The study initially included a total of 101,316 participants. Participants who did not attend, had unresolved status regarding diabetes or prediabetes (n=31,476), were under the age of 18 (n=12,606), or for whom the TyG index could not be calculated (n=31,852) were excluded. Participants with extreme values of the TyG index (mean ± 3 standard deviations) were also excluded (n=223). Ultimately, a total of 25,159 participants with complete data were incorporated into this analysis (**Figure 1**).

### 2.2 Calculation of TyG index

The formula for calculating the TyG index is as follows: TyG index = Ln [fasting triglycerides (mg/dL) x fasting glucose (mg/dL)/2] (25). This equation depicts the logarithmic conversion of the product of fasting triglyceride and glucose levels, divided by two. For our investigation, we utilized the TyG index as a continuous variable, which we later categorized into quartiles based on its values for further analysis. It’s crucial to clarify that within the scope of our study design, the TyG index is viewed as an exposure variable.

### 2.3 Assessment of the diagnosis of prediabetes and diabetes

Prediabetes is defined by any of the following criteria: (1) diagnosis by healthcare professionals, (2) a hemoglobin A1c (HbA1c) level between 5.7% and less than 6.5%, (3) a fasting plasma glucose (FPG) level between 5.6 mmol/L and 7.0 mmol/L, or (4) a 2-hour FPG value during an oral glucose tolerance test (OGTT) between 7.8 mmol/L and 11.0 mmol/L.

Diabetes is identified by meeting one or more of the following criteria: (1) a confirmed medical diagnosis by the patient’s healthcare providers, (2) a HbA1c level exceeding 6.5%, (3) an FPG level of 7.0 mmol/L or higher, (4) a random blood glucose level of 11.1 mmol/L or higher, or (5) a two-hour blood glucose level of 11.1 mmol/L or more following an OGTT.

### 2.4 Covariates

We assessed demographic variables, lifestyle factors, anthropometric measurements, and laboratory tests through computer-assisted personal interviews in this study. Demographic information included age, gender, ethnicity, socioeconomic status, and education level of individuals. Lifestyle factors encompassed smoking, drinking, physical activity, and diet (measured by the Healthy Eating Index (HEI)-2015). During physical health examinations, we considered blood pressure measurements, and laboratory tests specifically targeted the estimated glomerular filtration rate (eGFR). For this study, we categorized smoking status into three groups: never-smokers who had consumed fewer than 100 cigarettes in their lifetime, former smokers who had smoked over 100 cigarettes but had ceased by the time of the survey, and current smokers who had smoked more than 100 cigarettes and continued to do so regularly. Current drinking was divided into three categories: mild (≥3 drinks per day for women; ≥4 drinks per day for men; or binge drinking on 5 or more days per month), moderate (≥2 drinks per day for women; ≥3 drinks per day for men; or binge drinking at least twice a month), and heavy (all other cases). We classified a poverty income ratio below 1.3 as low, a ratio between 1.3 and 3.5 (inclusive) as medium, and a ratio above 3.5 as high. The assessment of physical activity levels involved calculating the metabolic equivalent of task (MET)/week by multiplying the total minutes spent on various activities each week by their respective metabolic equivalents, as estimated by the Compendium of Physical Activities. This methodology facilitated an accurate assessment of the intensity and frequency of an individual’s physical activity throughout the week. With the Compendium of Physical Activities providing standardized metabolic equivalents for different activities, this calculation was built on scientifically sound data, minimizing potential inaccuracies and bias. The physical activity level was quantified in terms of hours of activity per week (MET/week), and results were divided into three groups: low (<600 METs/week), moderate (600-1199 METs/week), and vigorous (≥1200 METs/week). The eGFR was calculated using the 2009 Serum Creatinine (SCr)-based CKD-EPI equation (26). The HEI-2015 is structured with a scoring range from 0 to 100, based on the aggregate of 13 distinct elements (27). Initially, it encompasses nine components focused on adequate consumption, which includes total fruits, whole fruits, total vegetables, greens and beans, total protein foods, as well as seafood and plant proteins, each scoring up to 5 points; and whole grains, dairy, and fatty acids, each valued up to 10 points. Additionally, it includes four moderation components: sodium, refined grains, added sugars, and saturated fats, each also valued up to 10 points. A higher score on the HEI-2015 reflects superior dietary quality. The calculation of these 13 components in the HEI-2015 is based on the comprehensive nutrient intake from the first day (DR1TOT), the second day (DR2TOT), and data from the United States Department of Agriculture (USDA) MyPyramid Equivalents Database/Food Patterns Equivalents Database (MPED/FPED).

### 2.5 Statistical analysis

We represented continuous variables using the mean and standard deviation (SD), while categorical variables were expressed as proportions. For variables that adhered to a normal distribution, we analyzed them using either the Student’s t-test or the chi-square test. We employed non-parametric tests or Fisher’s exact probability tests for variables with non-normal distributions. We used multivariable logistic regression analysis to explore the relationship between the TyG index and prediabetes and diabetes in the overall population. Model 1 presented raw data. Model 2 incorporated age, sex, race/ethnicity, and education level. In Model 3, we made additional adjustments to account for factors such as age, sex, race/ethnicity, education level, smoking, drinking, poverty income ratio, METs/week, systolic blood pressure (SBP), and eGFR. The logistic regression analysis provided odds ratios (ORs) alongside 95% confidence intervals (CIs). Taking into account the different birth cohort effects, we also carried out distinct analyses on different cycles as part of our sensitivity analysis. To confirm the consistency of this relationship, we conducted linear trend tests. In addition, we examined the dose-response relationship between the TyG index and prediabetes and diabetes using generalized additive models and fitting smooth curves. To assess potential interactions between the TyG index, prediabetes, and diabetes, we added interaction terms to the regression models and performed stratified analysis. We carried out statistical analyses using R (version 3.5.3) and EmpowerStats (http://www.EmpowerStats.com), considering a P-value below 0.05 as indicative of statistical significance.

## 3 Results

### 3.1 Baseline characteristics

A total of 25,159 participants were included in the study, composed of 12,164 males and 12,995 females. The average age of the participants was 47.59 years, with a standard deviation of 19.34. Additionally, the mean TyG index was 8.61 with a standard deviation of 0.64. Approximately 23.88% of individuals had prediabetes, while around 16.22% were diagnosed with diabetes. **Table 1** presents the demographic and clinical characteristics of the participants, categorized into quartiles according to their initial TyG index. All variables, except for METs/week and HEI, showed statistical significance across the four TyG groups. The group with the highest TyG index (TyG Q4) typically comprised older individuals, had a greater percentage of males, was predominantly Non-Hispanic Whites, and had lower levels of education. This group also exhibited a higher likelihood of being current or former smokers. Furthermore, compared to groups with lower TyG indexes, this group showed a higher frequency of past alcohol consumption, a lower poverty income ratio, increased blood pressure levels, and a reduced eGFR.

**Table 1:** Baseline characteristics of subjects.

Variables	TyG index quartiles				P-value
	Q1 (6.58-8.16) n=6289	Q2 (8.16-8.58) n=6288	Q3 (8.58-9.03) n=6290	Q4 (9.03-10.69) n=6292	
**Age (years)**	39.53 ± 18.33	47.11 ± 19.50	50.67 ± 18.94	53.30 ± 17.40	<0.001
**Sex (%)**					<0.001
Male	40.66	47.87	50.49	54.37	
Female	59.34	52.13	49.51	45.63	
**Race/ethnicity (%)**					<0.001
Non-Hispanic White	36.73	43.89	46.04	47.77	
Non-Hispanic Black	32.90	22.41	15.25	11.71	
Mexican American	13.45	17.03	19.97	23.66	
Others	16.92	16.67	18.74	16.85	
**Educational level (%)**					<0.001
Less than high school	22.79	26.59	29.97	33.65	
High school	22.98	24.17	23.77	24.09	
More than high school	54.24	49.24	46.26	42.26	
**Smoking (%)**					<0.001
Never	63.83	55.12	52.91	47.30	
Former	18.59	23.68	26.34	30.75	
Now	17.59	21.20	20.75	21.95	
**Drinking (%)**					<0.001
Never	15.75	14.05	14.04	15.72	
Former	12.32	16.23	19.19	22.09	
Mild	33.93	33.90	34.14	30.88	
Moderate	19.05	15.43	12.52	11.80	
Heavy	18.95	20.39	20.11	19.51	
**Poverty income ratio (%)**					<0.001
Low	31.50	30.96	31.21	32.80	
Medium	37.09	37.82	38.74	39.65	
High	31.41	31.22	30.06	27.55	
**METs/week (%)**					0.583
Low	95.17	95.23	95.05	95.37	
Moderate	2.51	2.77	2.97	2.77	
Vigorous	2.33	2.00	1.98	1.86	
**SBP (mmHg)**	117.20 ± 17.23	122.30 ± 19.03	124.66 ± 19.22	127.95 ± 19.50	<0.001
**DBP (mmHg)**	67.42 ± 11.36	69.00 ± 11.89	70.00 ± 12.28	71.00 ± 12.78	<0.001
**eGFR (ml/min/1.73 m^2^)**	105.43 ± 23.54	96.89 ± 24.21	93.46 ± 24.69	90.61 ± 25.10	<0.001
**HEI-2015**	49.93 ± 13.74	50.22 ± 13.62	50.38 ± 13.37	50.30 ± 13.30	0.282
**Glucose metabolism state (%)**					<0.001
None prediabetes	70.89	53.91	39.63	24.24	
Prediabetes	24.30	36.67	43.15	37.40	
Diabetes	4.82	9.41	17.22	38.37	

TyG, triglyceride-glucose; MET, metabolic equivalent of task; SBP, systolic blood pressure; DBP, diastolic blood pressure; eGFR, estimated glomerular filtration rate; HEI, healthy eating index.

### 3.2 Association between TyG index and prediabetes and diabetes

**Figure 2:**
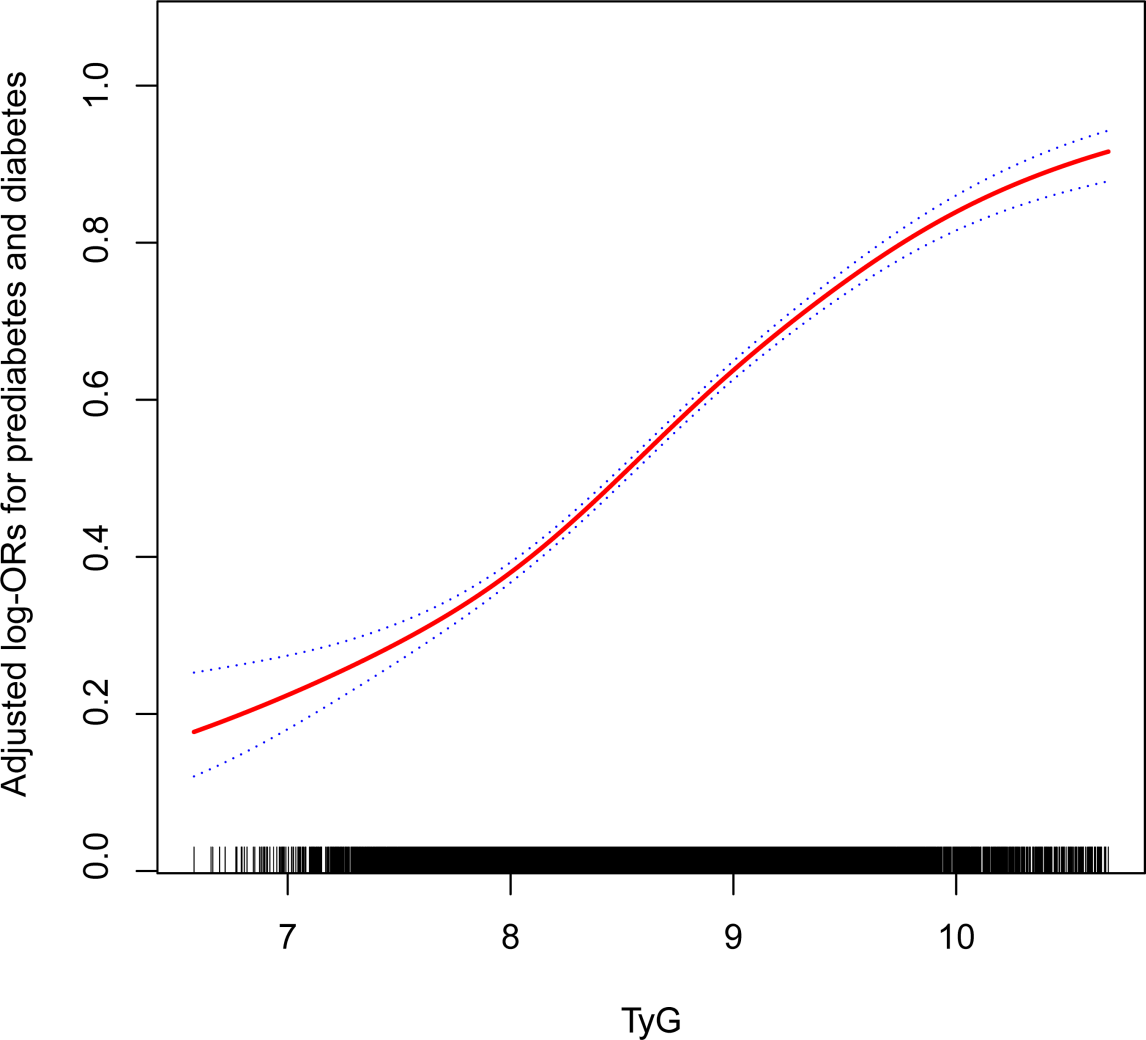
The association between TyG and the prevalence of prediabetes and diabetes. Age, sex, race/ethnicity, education level, smoking, drinking, poverty income ratio, METs/week, SBP, HEI-2015, and eGFR were adjusted. TyG, triglyceride-glucose; MET, metabolic equivalent of task; SBP, systolic blood pressure; HEI, healthy eating index; eGFR, estimated glomerular filtration rate.

**Table 2** exhibits the logistic regression model results, illustrating the association between the TyG index and prediabetes and diabetes. A significant positive association between the TyG index and both prediabetes and diabetes was observed in all participants, irrespective of whether covariates were adjusted. The TyG index was divided into quartiles, with Group Q1 utilized as the reference for evaluating the relationship between the TyG index and prediabetes and diabetes. After controlling for age, sex, race/ethnicity, education level, smoking, drinking, poverty income ratio, METs/week, SBP, HEI-2015, and eGFR, the relative odds of prediabetes and diabetes for participants in the Q2 (OR: 1.69; 95%CI: 1.52, 1.89), Q3 (OR: 2.57; 95%CI: 2.30, 2.88), and Q4 (OR: 4.88; 95%CI: 4.33, 5.49) groups demonstrated a linear increase compared to the reference group, Q1. This observation was further substantiated by a significant P-value for trend of <0.001, indicating a clear positive association between the TyG index and the occurrence of prediabetes and diabetes (**Table 2**). During the examination of how TyG index relates to prediabetes and diabetes across various cycles, it was also observed that as TyG levels rise, the risk of prediabetes and diabetes incrementally increases (**Table S1**). Meanwhile, the outcomes from fitting a smooth curve are comparable to the results obtained through multiple regression analysis (**Figure 2**).

**Table 2:** Relationship between TyG index and prediabetes and diabetes in different models.

TyG index	Model 1	Model 2	Model 3
**Continuous**	3.54 (3.37, 3.71)	2.95 (2.80, 3.11)	2.81 (2.62, 3.01)
Quartiles			
Q1 (6.58-8.16)	Reference	Reference	Reference
Q2 (8.16-8.58)	2.02 (1.88, 2.17)	1.63 (1.50, 1.76)	1.69 (1.52, 1.89)
Q3 (8.58-9.03)	3.50 (3.25, 3.77)	2.66 (2.45, 2.89)	2.57 (2.30, 2.88)
Q4 (9.03-10.69)	7.25 (6.69, 7.85)	5.34 (4.89, 5.84)	4.88 (4.33, 5.49)
**P for trend**	<0.001	<0.001	<0.001

Model 1: Non-adjusted.

Model 2: Adjusted for age, sex, race/ethnicity and education level.

Model 3: Adjusted for age, sex, race/ethnicity, education level, smoking, drinking, poverty income ratio, METs/week, SBP, HEI-2015, and eGFR.

TyG, triglyceride-glucose; MET, metabolic equivalent of task; SBP, systolic blood pressure; HEI, healthy eating index; eGFR, estimated glomerular filtration rate.

The outcomes of the two-piecewise logistic regression model suggest a potential non-linear relationship between the TyG index and prediabetes and diabetes, with an inflection point at 8.00. For individuals with a TyG index less than 8.00, the adjusted OR between the TyG index and prediabetes and diabetes was 1.80 (95% CI: 1.33 2.45); for those with a TyG index greater than 8.00, the adjusted OR was 3.00 (95% CI: 2.76, 3.27). The log-likelihood ratio test revealed a statistically significant difference (P=0.004) between the two slopes for values above and below 8.00 (**Table 3**). Furthermore, this analysis also highlights gender-based differences in the inflection points: for males, it aligns with the overall population at 8.00, but for females, it shifts to 9.00 (**Tables** **S2, S3**).

**Table 3:** The result of two-piecewise logistic regression model.

TyG index	Adjusted OR^*^ (95% CI)	P-value
Model I		
Fitting by the standard linear model	2.81 (2.62, 3.01)	<0.001
Model II		
Inflection point	8.00	
< 8.00	1.80 (1.33, 2.45)	0.002
> 8.00	3.00 (2.76, 3.27)	<0.001
**Log likelihood ratio**	/	0.004

^*^Adjusted for age, sex, race/ethnicity, education level, smoking, drinking, poverty income ratio, METs/week, SBP, HEI-2015, and eGFR.

TyG, triglyceride-glucose; MET, metabolic equivalent of task; SBP, systolic blood pressure; HEI, healthy eating index; eGFR, estimated glomerular filtration rate.

**Figure 3:**
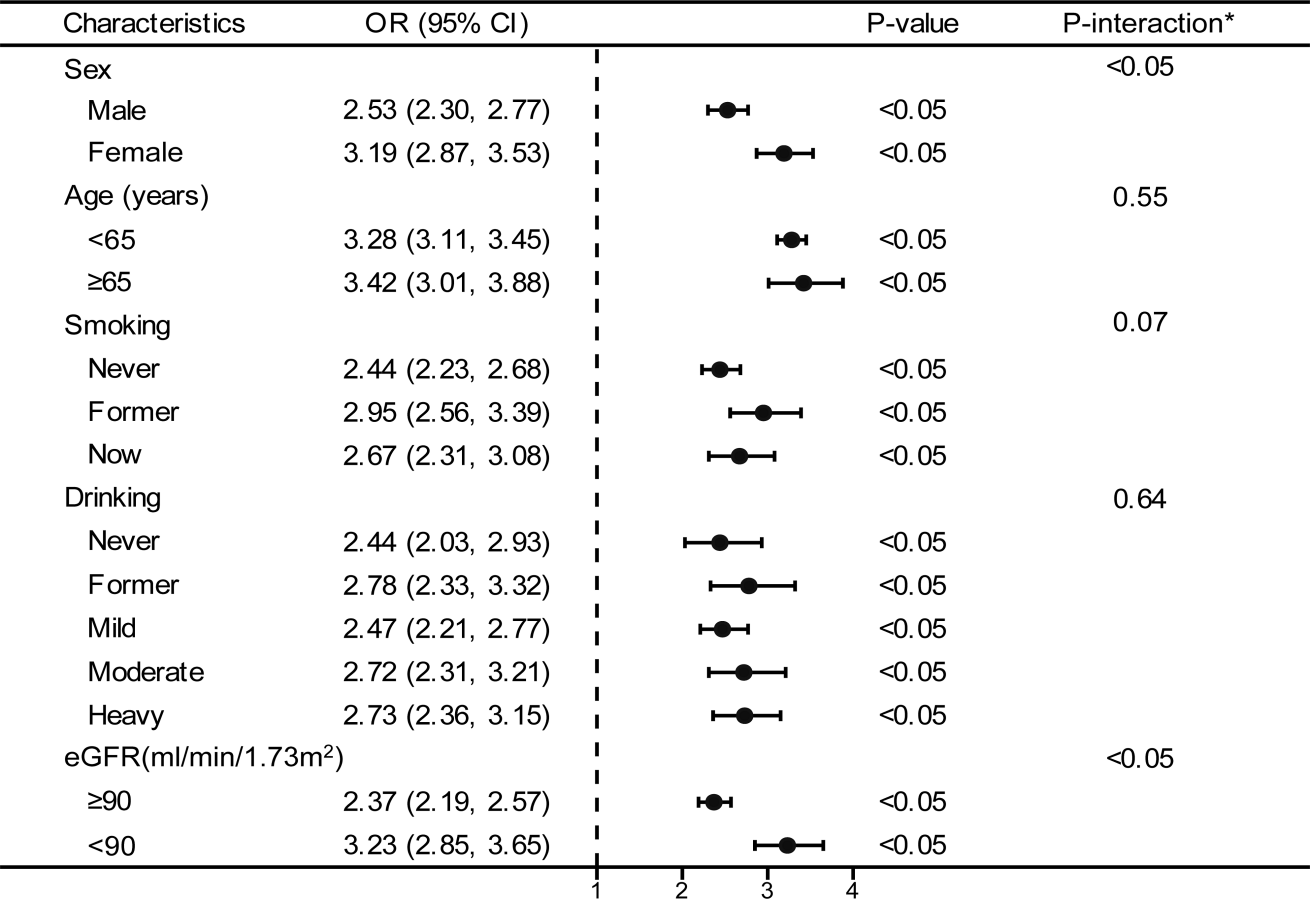
Stratified analyses between TyG and the prevalence of prediabetes and diabetes. *Each stratification adjusted for all the factors (age, sex, race/ethnicity, education level, smoking, drinking, poverty income ratio, METs/week, SBP, HEI-2015, and eGFR) except the stratification factor itself. OR, odd ratio; CI, confidence interval; TyG, triglyceride-glucose; MET, metabolic equivalent of task; SBP, systolic blood pressure; HEI, healthy eating index; eGFR, estimated glomerular filtration rate.

**Figure 4:**
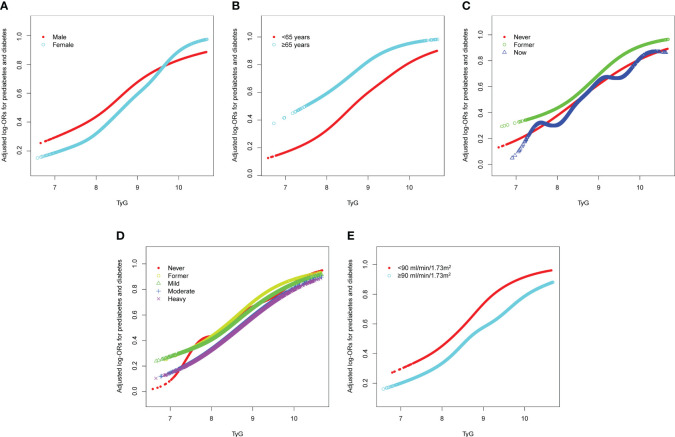
Stratified analyses [by **(A)** sex; **(B)** age; **(C)** smoking; **(D)** drinking; **(E)** eGFR] between TyG and the prevalence of prediabetes and diabetes using generalized additive model and smooth curve fittings. Each generalized additive model and smooth curve fitting was adjusted for all factors, including age, sex, race/ethnicity, education level, smoking, drinking, poverty income ratio, METs/week, SBP, HEI-2015, and eGFR, except for the stratification factor itself. TyG, triglyceride-glucose; MET, metabolic equivalent of task; SBP, systolic blood pressure; HEI, healthy eating index; eGFR, estimated glomerular filtration rate.

The forest plot indicated a significant interaction between the TyG index and the presence of prediabetes and diabetes regarding gender and eGFR (P<0.05). Moreover, we observed a direct relationship between the TyG index and the incidence of prediabetes and diabetes in all classifications (as shown in **Figure 3**). To enhance sensitivity of our analysis, we carried out a stratified analysis on smooth curve fitting to examine the association between the TyG index and both prediabetes and diabetes. **Figure 4** illustrated a positive association between these variables, regardless of gender, age (over or under 65 years), smoking habits (never, former, and now), alcohol consumption (never, former, mild, moderate, and heavy), and eGFR (over or under 90 ml/min/1.73 m²).

## 4 Discussion

In this study involving U.S. adults, we observed a positive association between the TyG index and risk indicators for prediabetes and diabetes, even after adjusting for confounding variables. Compared to the group with the lowest TyG index quartile, the group with the highest TyG index quartile was 4.88 times more likely to have prediabetes and diabetes. Additionally, a non-linear relationship with an inflection point of 8.00 was identified between the TyG index and prediabetes and diabetes. These results suggest that the TyG index could potentially serve as a monitoring marker for prediabetes and diabetes.

Previous studies have explored the relationship between the TyG index and diabetes, with all demonstrating a positive association between the TyG index and diabetes. A meta-analysis of 15 cohort studies highlighted a significant positive association between the TyG index and the risk of Type 2 Diabetes (T2D), suggesting that the TyG index can be a useful tool for identifying individuals at risk of T2D (28). Different researchers have also investigated the impact of age distribution. A study from South Korea revealed that the TyG index was significantly associated with insulin resistance in T2D and was more effective than Homeostatic Model Assessment of Insulin Resistance (HOMA-IR) in predicting T2D in children and adolescents (29). Chen et al. found a multivariable Hazard Ratio of 1.22 (95% CI, 1.14-1.31) for each SD increase in the TyG index among 7,428 participants in a Chinese adult study (30). They observed a higher multivariable adjusted hazard ratio in the female population and in individuals over 65 years in the subgroup analysis. This aligns with the findings of our study, suggesting the TyG index plays a role in predicting diabetes, particularly when considering common risk factors. However, prediabetic patients, who are at high risk of developing diabetes, have generally received less attention. Only recently have researchers begun to study the association between the TyG index and prediabetes. Prediabetes, an asymptomatic chronic moderate hyperglycemic state, can progress to diabetes if left undetected (31). Therefore, in our study, we considered prediabetes and diabetes together as outcome variables to examine their relationship with the TyG index. This cross-sectional analysis found a 4.88-fold increased risk of prediabetes and diabetes in the group with the highest TyG index compared to the group with the lowest TyG index. Simultaneously, the positive association was not impacted by factors such as gender, age, smoking, drinking, and eGFR. There was a stable positive association between the TyG index and prediabetes and diabetes.

In our analysis, we found a possible non-linear relationship between the TyG index and prediabetes and diabetes. Xuan et al. suggested a potential U-shaped association between baseline TyG index and the risk of developing diabetes in a Japanese population with normal glucose levels (18). This is inconsistent with our study’s findings. In the study by Xuan et al., only diabetes was included in the outcome measures. Due to the limited duration of follow-up, most pre-diabetic patients who were likely to develop diabetes in the future were excluded. Simultaneously, their large sample study lacked an oral glucose tolerance test when collecting outcome measures, possibly underestimating the incidence of diabetes. Given this, it is essential to validate the non-linear relationship in the U.S. population with the combination of prediabetes and diabetes as outcome variables. In line with our findings, Li et al. discovered a significant non-linear relationship between the TyG index and future diabetes risk after adjusting for covariates (32). However, their study only explored the non-linear relationship using a generalized additive model and did not conduct further two-piecewise cox regression to determine the exact inflection point. Our results suggest that the adjusted OR between the TyG index and prediabetes and diabetes is 1.7 times as high for patients with a TyG index greater than 8.00, as compared to those with a TyG index less than 8.00. A dose-response meta-analysis of the TyG index and diabetes demonstrated that the dose-response curve steepens when the TyG index exceeds 8.6 (33). This may be attributed to some overlapping sample sizes due to the use of the DataDryad database in this meta-study, which gives more weight to studies of the East Asian population, thus potentially introducing some bias into the results. Our research indicates that the inflection point differs across genders compared to the general population trend. For female participants, this critical value is identified at a TyG index of 9.00, while it is 8.00 for males. This variation might stem from several gender-specific factors. Women generally possess a higher body fat percentage and are subject to more substantial metabolic alterations, particularly during hormonal changes such as menopause (34). Moreover, the fluctuation in sex hormone levels could play a role in modifying the risk of prediabetes and diabetes at various TyG index values (35). Additionally, the difference in dietary habits and physical activity routines between the sexes could have a more pronounced impact on their metabolic health and diabetes risk (36).

We conducted a stratified analysis to examine the effect of the TyG index separate from the previously mentioned covariates. Interestingly, the results of the forest plot-based logistic regression and subgroup analysis of the generalized additive models demonstrated that the positive association between the TyG index and both prediabetes and diabetes remained robust regardless of sex, age, smoking, drinking, and eGFR. This confirms the reliability and generalizability of our results. Additionally, sex and eGFR appeared to impact the association between the TyG index and prediabetes and diabetes (significant P value for interaction). It is commonly hypothesized that the higher risk of diabetes in women in later adulthood is due to changes in menopausal hormones, such as the decrease in estrogen (37, 38). Epidemiological studies have shown that women are at an increased risk of developing diabetes and its associated complications as they age (39, 40), suggesting that greater attention should be paid to women when considering the predictive significance of the TyG index for prediabetes and diabetes. In our study, we found that the TyG index predicted a higher risk of prediabetes and diabetes in individuals with an eGFR<90 ml/min/1.73 m^2^. In a study that tracked 1,713 Americans without diabetes but with reduced GFR, it was found that the incidence of T2D was significantly higher among individuals with chronic kidney disease (CKD) compared to the general population (41). This result was corroborated by another cohort study conducted in Taiwan, which singled out CKD as a distinct and significant predictor for diabetes (42). Mo et al. also found that eGFR was independently associated with the onset of diabetes, with a 1.4% reduction in diabetes risk for each 1 mL/min·1.73 m^2^ increase in eGFR (43). Therefore, as the interaction analysis in this study showed, individuals with impaired renal function have a higher risk of prediabetes and diabetes as predicted by the TyG index.

This study carries both strengths and limitations. A significant advantage is the large sample size drawn from the NHANES database, which uses a complex weighting design and is highly representative of the overall U.S. population. We used NHANES data spanning from 1999 to 2018. Secondly, this study recognizes prediabetes as an asymptomatic, chronic, moderately hyperglycemic state that warrants inclusion in diabetes prevention efforts. Moreover, we were the first to perform a thorough analysis of the non-linear relationship between the TyG index and prediabetes and diabetes using a smoothed fitting curve and two-part logistic regression.

However, the results need to be interpreted with caution due to several limitations. As a cross-sectional observational study, causality and directionality cannot be established. Even though we extensively adjusted for confounding factors, other potential influences cannot be completely ruled out. Additionally, the non-linear relationship between the TyG index and prediabetes and diabetes remains a contentious topic in numerous studies. Therefore, future longitudinal studies should be conducted to provide stronger evidence to substantiate the relationship between the TyG index and prediabetes and diabetes.

## 5 Conclusion

Our study, involving 25,159 participants, demonstrated a significant association between the TyG index and the increased risk of prediabetes and diabetes. We found that higher TyG index values are associated with a greater likelihood of these conditions, a relationship that remains consistent across different demographic and lifestyle groups. Notably, our research identified a non-linear relationship with an inflection point at a TyG index of 8.00, indicating varying risk intensities at different index values. These findings highlight the TyG index as a valuable marker for predicting the risk of prediabetes and diabetes, suggesting its potential role in healthcare for risk assessment and in guiding preventive strategies.

## Data availability statement

The raw data supporting the conclusions of this article will be made available by the authors, without undue reservation.

## Ethics statement

All data came from NHANES, which was approved by National Centre for Health Statistics Institutional Ethics Review Board, and all the subjects agreed on the survey and signed written consent. The studies were conducted in accordance with the local legislation and institutional requirements. The participants provided their written informed consent to participate in this study.

## Author contributions

LHZ: Conceptualization, Data curation, Formal Analysis, Investigation, Validation, Writing – original draft. LZ: Funding acquisition, Methodology, Project administration, Resources, Software, Supervision, Visualization, Writing – review & editing.
