# Supplementary material for: Non-linear association of triglyceride-glucose index with prevalence of prediabetes and diabetes: a cross-sectional study
Source: Front Endocrinol (Lausanne). 2023 Dec 13;14:1295641. doi: 10.3389/fendo.2023.1295641 (PMC10751584; doi:10.3389/fendo.2023.1295641)
Supplement: Supplementary file 1 [file DataSheet_1.docx]

**Table S1. Relationship between TyG index (as continuous variable) and prediabetes and diabetes in different cycles**

| **Cycles** | **Model 1** | **Model 2** | **Model 3** |
| --- | --- | --- | --- |
| **1999-2000** | 5.02 (4.21, 6.00) | 4.19 (3.42, 5.13) | 3.88 (2.89, 5.20) |
| **2001-2002** | 4.72 (4.02, 5.54) | 4.12 (3.45, 4.92) | 3.85 (3.02, 4.91) |
| **2003-2004** | 5.04 (4.26, 5.98) | 4.34 (3.60, 5.23) | 3.83 (2.97, 4.95) |
| **2005-2006** | 3.31 (2.84, 3.85) | 2.91 (2.44, 3.46) | 2.64 (2.10, 3.32) |
| **2007-2008** | 3.90 (3.34, 4.55) | 3.05 (2.58, 3.62) | 2.99 (2.38, 3.76) |
| **2009-2010** | 5.04 (4.28, 5.94) | 3.93 (3.29, 4.70) | 3.96 (3.08, 5.09) |
| **2011-2012** | 4.40 (3.73, 5.21) | 3.85 (3.20, 4.64) | 3.70 (2.88, 4.75) |
| **2013-2014** | 4.46 (3.83, 5.21) | 4.00 (3.37, 4.73) | 3.50 (2.82, 4.34) |
| **2015-2016** | 3.91 (3.32, 4.60) | 3.11 (2.60, 3.72) | 2.80 (2.23, 3.52) |
| **2017-2018** | 4.18 (3.54, 4.94) | 3.20 (2.67, 3.83) | 3.43 (2.63, 4.48) |

**Model 1:** Non-adjusted.

**Model 2:** Adjusted for age, sex, race/ethnicity and education level.

**Model 3:** Adjusted for age, sex, race/ethnicity, education level, smoking, drinking, poverty income ratio, METs/week, SBP, HEI-2015, and eGFR.

**Abbreviations:** TyG, triglyceride-glucose; MET, metabolic equivalent of task; SBP, systolic blood pressure; HEI, healthy eating index; eGFR, estimated glomerular filtration rate.

**Table S2.** **The result of two-piecewise logistic regression model in males**

| **TyG index** | **Adjusted OR^*^ (95% CI)** | **P-value** |
| --- | --- | --- |
| **Model I** |  |  |
| Fitting by the standard linear model | 2.46 (2.24, 2.70) | <0.001 |
| **Model II** |  |  |
| Inflection point | 8.00 |  |
| < 8.00 | 1.53 (0.97, 2.41) | 0.065 |
| > 8.00 | 2.61 (2.34, 2.92) | <0.001 |
| **Log likelihood ratio** | / | 0.039 |

**Notes:** ^*^Adjusted for age, race/ethnicity, education level, smoking, drinking, poverty income ratio, METs/week, SBP, HEI-2015, and eGFR.

**Abbreviations:** TyG, triglyceride-glucose; MET, metabolic equivalent of task; SBP, systolic blood pressure; HEI, healthy eating index; eGFR, estimated glomerular filtration rate.

**Table S3.** **The result of two-piecewise logistic regression model in females**

| **TyG index** | **Adjusted OR^*^ (95% CI)** | **P-value** |
| --- | --- | --- |
| **Model I** |  |  |
| Fitting by the standard linear model | 3.35 (3.01, 3.74) | <0.001 |
| **Model II** |  |  |
| Inflection point | 9.00 |  |
| < 9.00 | 2.95 (2.55, 3.41) | <0.001 |
| > 9.00 | 5.20 (3.63, 7.45) | <0.001 |
| **Log likelihood ratio** | / | 0.009 |

**Notes:** ^*^Adjusted for age, sex, race/ethnicity, education level, smoking, drinking, poverty income ratio, METs/week, SBP, HEI-2015, and eGFR.

**Abbreviations:** TyG, triglyceride-glucose; MET, metabolic equivalent of task; SBP, systolic blood pressure; HEI, healthy eating index; eGFR, estimated glomerular filtration rate.
